# Changes in hormone flux and signaling in white spruce (*Picea glauca*) seeds during the transition from dormancy to germination in response to temperature cues

**DOI:** 10.1186/s12870-015-0638-7

**Published:** 2015-12-18

**Authors:** Yang Liu, Kerstin Müller, Yousry A. El-Kassaby, Allison R. Kermode

**Affiliations:** Department of Forest and Conservation Sciences, Faculty of Forestry, University of British Columbia, Vancouver, British Columbia V6T 1Z4 Canada; Department of Biological Sciences, Simon Fraser University, Burnaby, British Columbia V5A 1S6 Canada

**Keywords:** Seed dormancy, Auxin, ABA, GAs, Moist-chilling, Seed germination, White spruce

## Abstract

**Background:**

Seeds use environmental cues such as temperature to coordinate the timing of their germination, allowing plants to synchronize their life history with the seasons. Winter chilling is of central importance to alleviate seed dormancy, but very little is known of how chilling responses are regulated in conifer seeds. White spruce (*Picea glauca*) is an important conifer species of boreal forests in the North American taiga. The recent sequencing and assembly of the white spruce genome allows for comparative gene expression studies toward elucidating the molecular mechanisms governing dormancy alleviation by moist chilling. Here we focused on hormone metabolite profiling and analyses of genes encoding components of hormone signal transduction pathways, to elucidate changes during dormancy alleviation and to help address how germination cues such as temperature and light trigger radicle emergence.

**Results:**

ABA, GA, and auxin underwent considerable changes as seeds underwent moist chilling and during subsequent germination; likewise, transcripts encoding hormone-signaling components (e.g. ABI3, ARF4 and Aux/IAA) were differentially regulated during these critical stages. During moist chilling, active IAA was maintained at constant levels, but IAA conjugates (IAA-Asp and IAA-Glu) were substantially accumulated. ABA concentrations decreased during germination of previously moist-chilled seeds, while the precursor of bioactive GA1 (GA53) accumulated. We contend that seed dormancy and germination may be partly mediated through the changing hormone concentrations and a modulation of interactions between central auxin-signaling pathway components (TIR1/AFB, Aux/IAA and ARF4). In response to germination cues, namely exposure to light and to increased temperature: the transfer of seeds from moist-chilling to 30 °C, significant changes in gene transcripts and protein expression occurred during the first six hours, substantiating a very swift reaction to germination-promoting conditions after seeds had received sufficient exposure to the chilling stimulus.

**Conclusions:**

The dormancy to germination transition in white spruce seeds was correlated with changes in auxin conjugation, auxin signaling components, and potential interactions between auxin-ABA signaling cascades (e.g. the transcription factor ARF4 and ABI3). Auxin flux adds a new dimension to the ABA:GA balance mechanism that underlies both dormancy alleviation by chilling, and subsequent radicle emergence to complete germination by warm temperature and light stimuli.

**Electronic supplementary material:**

The online version of this article (doi:10.1186/s12870-015-0638-7) contains supplementary material, which is available to authorized users.

## Background

Conifers are ecologically and economically important plants, and coniferous forests cover vast tracts in the Northern hemisphere. White spruce (*Picea glauca*) is a keystone species of boreal forests in the North American taiga. In Canada, over 100 million white spruce seedlings are out-planted yearly for regeneration [1]. However, our understanding of molecular mechanisms underlying the dormancy and germination of white spruce seeds and of conifers in general remains quite limited. As the white spruce genome was the first to be sequenced and assembled amongst conifer species in 2013, interest in investigating aspects of the molecular mechanisms underlying key developmental and physiological processes is mounting [2–4].

Moist-chilling is a common dormancy-breaking stimulus for conifer seeds both in natural stands and under laboratory conditions. Specific requirements can vary enormously amongst different conifer species, as well as amongst different clones and seed lots of a given species [5, 6]; for white spruce, the typical moist-chilling requirement under laboratory conditions is approximately 21 days.

During seed maturation, exposure of seeds on the parent plant to low temperatures can influence the depth of primary dormancy of the mature seeds. In the imbibed mature dormant seed, dormancy alleviation is often promoted by exposure to chilling. It is therefore assumed that chilling plays a dual role in regulating dormancy [7]. In addition, under some conditions, extended chilling can result in secondary dormancy [8, 9]. Mechanisms that underlie the beneficial effects of moist-chilling on dormancy alleviation undoubtedly involve plant hormones - with abscisic acid (ABA) and gibberellins (GAs) receiving the most attention, alone and within the context of their interplay or crosstalk with other hormones such as auxins, cytokinins, and ethylene [10–14].

Although evolutionarily independent from the other seed-bearing plants since 260 million years ago [15], the seeds of conifers exhibit conserved mechanisms regulating their dormancy and germination with seeds of angiosperms, including those mediated by ABA [16–18]. Several studies demonstrate that moist-chilling invokes changes in the levels of, and sensitivity to, ABA and GAs in conifer seeds [19–21]. ABA levels are reduced during moist-chilling-induced dormancy termination of yellow-cypress (*Callitropsis nootkatensis*) and Douglas-fir (*Pseudotsuga menziesii*) seeds [22, 23]. ABA levels of western white pine (*Pinus monticola*) seeds also decline significantly during moist-chilling, and this decline is associated with an increase in germination capacity [24]. It is noteworthy that if dormancy-breaking conditions are not met, seeds maintain high ABA levels; and dormancy imposition and maintenance require ABA biosynthesis [25]. For western white pine seeds, it is the ratio of ABA biosynthesis to catabolism that appears to be the key factor that determines the capacity for dormancy maintenance versus germination. GAs have a positive effect on dormancy alleviation and germination of conifer seeds [25]; likewise, dormancy alleviation of moist-chilled Arabidopsis seeds depends on the expression of GA_3_ oxidase 1 of the GA biosynthesis pathway [19, 21]. In hazel (*Corylus avellana*), moist-chilling has a pronounced effect on the capacity of the seeds for GA biosynthesis, although active GA production does not take place until the seeds are placed in germination conditions [26].

In the ABA signalling cascade of Arabidopsis, concerted actions of four transcription factors, i.e. ABSCISIC ACID INSENSITIVE 3 (ABI3), FUSCA 3 (FUS3), LEAFY COTYLEDON 1 (LEC1), and LEAFY COTYLEDON 2 (LEC2), mediate various seed maturation processes and some of these factors also participate in the transition from dormancy to germination [27, 28]. Orthologs of *ABI3*, encoding a structurally conserved transcription factor have been isolated from angiosperm and gymnosperm (conifer) species, and they act as central regulators of seed development and dormancy [10, 29]. A member of the *ABI3*/*VP1* family cloned from yellow-cypress is positively associated with dormancy maintenance [30]. Through yeast two-hybrid analyses, a yellow-cypress ABI3 Interacting protein (CnAIP2) that functions as a negative regulator of ABI3 was recently identified [31]; note that this protein is different from the Arabidopsis E3 ubiquitin ligase, AIP2 [32]. CnAIP2, like CnABI3, acts a central gatekeeper of important plant life cycle transitions including the seed dormancy-to-germination transition [31].

GAs also modulate plant growth and development and can act antagonistically to ABA in the control of both seed dormancy and germination [11, 33]. Notably, regulation of seed germination via light and temperature is correlated with GA metabolism and signalling in many species [10, 19, 27, 34]. Exogenous application of GAs to western white pine seeds initiates a decrease in ABA levels in dormant seeds by changing ABA homeostasis, i.e. promoting ABA catabolism or transport over ABA biosynthesis [25].

The hormone auxin (principally indole-3-acetic acid [IAA]) regulates many aspects of plant growth and development. Amide-linked conjugates of IAA synthesized during seed development [35, 36] can serve as a source of free IAA during seed germination [37, 38]. Several lines of evidence implicate a role for auxins in seed dormancy maintenance in Arabidopsis [39–41]; auxin-mediated seed dormancy maintenance depends on ABI3 and this inhibitory effect can be nullified by moist-chilling [42]. The hub of the auxin signalling pathway is the TRANSPORT INHIBITOR RESPONSE1 (TIR1)/AUXIN SIGNALING F-BOX (AFB) proteins signaling system [43–45].

In this work, we studied one white spruce (*Picea glauca*) population from British Columbia, Canada, to elucidate the hormone-based mechanisms that underpin dormancy alleviation and germination in response to temperature signalling (i.e. moist chilling and transfer to germination conditions). This research will help provide insights into how winter chilling contributes to the timing of phenology, and how conifer life histories may develop under new climate scenarios.

## Methods

### Seed materials, germination testing, and seed sampling

One white spruce population from British Columbia, Canada (located at 54°26’N, 121°44’W, 850 m elevation), was selected for this study based on cumulative germination performance after the standard 21-day moist-chilling treatment [46]. For germination characterization, seeds were first moist-chilled in clear plastic germination boxes (Hoffman) lined with moistened cellulose wadding and filter paper, and moistened with 50 mL of sterile water for 21 days at 3 °C in a dark environment. The boxes containing seeds were then transferred into germination conditions (30/20 °C, 8-h-photoperiod and 70 % relative humidity). Light was provided by fluorescence illumination at approximately 13.5 μmol · m^−2^s^−1^. Standard germination was conducted over a 21-day span following the International Seed Testing Association standards [47]. As controls for the transfer to the germination-promoting conditions (30/20 °C and light), seeds were transferred to constant darkness at 30/20 °C, or were kept in moist-chilling conditions (constant 3 °C) with an 8-h photoperiod. Germination assays, scoring, and quantification were performed as previously described [46].

**Fig. 1:**
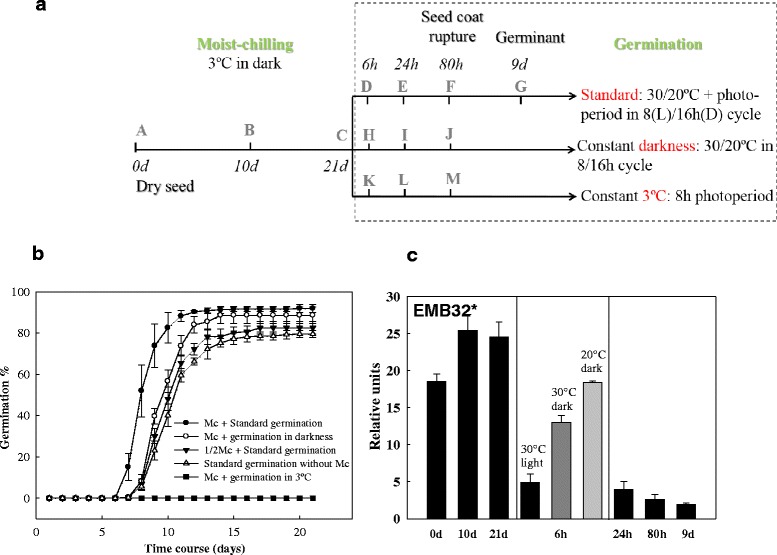
Effect of moist chilling on the germination performance of white spruce seeds. **a** Schematic representation of sampling to determine germination of white spruce seeds in different germination conditions with a 21-d or 10-d moist-chilling period (Mc or 1/2Mc) or no moist chilling. In standard germination conditions (30/20 °C and an 8-h photoperiod), seed coat rupture and radicle protrusion was observed at 80 h in the majority of the population. The stage at which the radicle had emerged to four times of the seed length was reached on d 9. **b** Germination of white spruce seeds under the conditions represented in (**a**) and without moist chilling. Data points are means ± SE of four dishes of 100 seeds each. While biologists define the completion of germination as radicle emergence, ‘germination’ percentage in the forest industry is based on the number of seeds that reach the stage when the radicle has emerged to four times the seed length (approximately 4 mm for white spruce). In **b**, we used this latter definition. **c** Transcript dynamics of the dormancy marker, *EMB32* during moist-chilling (0, 10, 21 d), germination (6, 24, 80 h) and seedling growth (9 d) (black bars). At the 6 h time-point, transcript levels were determined under three conditions: in seeds after their transfer to standard germination conditions (30/20 °C and 8-h photoperiod) (black bar), in seeds maintained in darkness at 30 °C (dark grey bars), and in seeds maintained in darkness at 20 °C (light grey bars). Relative expression levels as determined by RT-qPCR are shown. Each data point is the average of three biological replicates. Bars indicate the SEM. Note: one asterisk (*) indicates that the gene has been annotated in gymnosperms but not in white spruce

Seed sampling for molecular and biochemical analyses was conducted on 3 biological replicates and included times during moist-chilling (0, 10 and 21 d) and after transfer to germination or control conditions (6, 24, 80 h, and 9 d) (Fig. 1a). For seeds that had been maintained in darkness, the sampling was also conducted in darkness. Samples comprising the 3 replicates were collected and immediately frozen in liquid N_2_ and stored at −80 °C.

### Reference gene selection and gene query using BLASTN

**Fig. 2:**
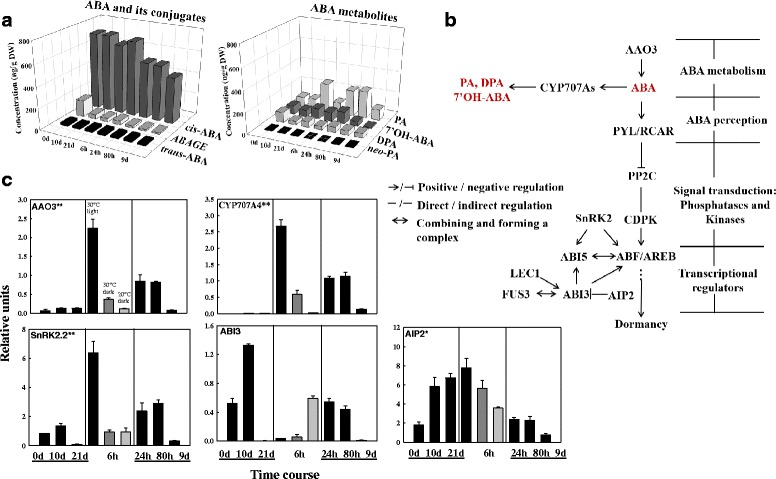
Changes in ABA, ABA metabolites, and ABA signalling components during the transition from dormancy to germination of white spruce seeds. **a** Profiles of ABA and its metabolites as determined by UPLC/ESI-MS/MS during moist-chilling at 3 °C (0, 10, and 21 days) and during germination (6, 24 and 80 h) and seedling growth (9 d). Each data point is the average of two biological replicates. **b** Schematic of ABA biosynthesis, signalling, and catabolism. **c** Transcript levels of ABA metabolism and signalling genes and selected downstream targets during moist-chilling (0, 10, 21 d), germination (6, 24, 80 h) and seedling growth (9 d) (black bars). Also shown are previously chilled seeds placed in two control treatments - 6 h germination conditions under darkness at 30 °C (dark grey bar) or 20 °C (light grey bar). Each data point is the average of three biological replicates. Bars indicate the SEM. Note: two superscript asterisks (**) indicate that the gene is only annotated in angiosperms; one asterisk (*) indicates that the gene has been annotated in gymnosperms but not in white spruce; no asterisk indicates that the gene has been annotated in white spruce

Three genes were chosen and used as internal controls: CO220221 (peroxisomal targeting signal receptor), CO206996 (hypothetical protein), and AY639585 (ubiquitin conjugating enzyme 1, UBC1); these were selected due to their constitutive expression during developmental transitions as determined by published microarray profiling [48, 49]. A subset of genes specifying proteins mediating the committed steps of ABA, GA and auxin biosynthesis/catabolism or signalling pathways (Fig. 2), were used to query the spruce EST database (PlantGDB) and the white spruce whole genome data (NCBI) using BLASTN. Primers (Additional file 1: Table S1) were designed with the primer3 tool online [50].

### RNA isolation, quantitative (q)RT-PCR and principle component analysis

RNA was isolated from seeds as previously outlined [51]. Two μg of RNA was reverse-transcribed into cDNA using the EasyScript Plus™ kit (abmGood) with oligo-dT primers. First-strand cDNA synthesis products were diluted fivefold, and one μl of cDNA was used to carry out semi-quantitative RT-PCR for a primer specificity check. Quantitative RT-PCR (qRT-PCR) analyses were run with three biological replicates per sample in 15-μl reaction volumes in an ABI7900HT machine (Applied Biosystems) using the PerfeCTa® SYBR® Green SuperMix with ROX (Quanta Biosciences). The reaction mixture consisted of 1.0 μl fivefold diluted cDNA, 7.5 μl supermix and 1.0 μl of each primer (10 μmol · L^−1^). The reaction procedure was 5 min at 95 °C, 45 cycles of 15 s at 95 °C and 60 s at 59 °C. Dissociation curves were generated at the end of each qRT-PCR to validate the amplification of only one product. Efficiency calculation and normalization were performed using real-time PCR Miner (www.miner.ewindup.info/) [52] and data quality was confirmed through internal controls and no-template-controls, and by comparing the repeatability across replicates. An average expression value for each gene at each time point was generated from the normalized data.

Principle component analysis (PCA) was performed using SAS® (vers. 9.3; SAS Institute Inc., Cary, NC) based on the expression patterns of all genes in different germination conditions at 6, 24, and 80 h as described in the text.

### Western blot analysis

Protein extracts were generated by grinding the seed materials in protein extraction buffer (50 mM Tris pH 8.0, 150 mM NaCl, 1 % Triton X-100, and 100 μg · ml^−1^ phenylmethylsulfonyl fluoride) and protein concentration was determined by measuring OD_750_ with the aid of photometer. Protein extracts (30 μg total soluble protein) were separated by 10 % SDS-PAGE and transferred onto Amersham Hybond-P (PVDF) membranes using wet electro-blotting. The blots were blocked overnight at 3 °C using 5 % (w/v) non-fat dry milk and 0.1 % (v/v) Tween-20 (PBST) followed by three washes (15 min each) with PBST. Blots were incubated with the anti-*pg*KS (*ent*-kaurene synthase) antibody (1:500 dilution) for 1 h at room temperature (provided by T-P. Sun’s lab). After three washes with PBST (15 min each) the membrane was incubated with the anti-rat HRP (horseradish peroxidase) antibody (1:40,000 dilution) for 1 h at room temperature. After three washes with PBST (15 min each) the membrane was drained and placed within wrap film containing 2 ml Supersignal West Pico solution and the membrane exposed to light. Chemiluminescent images were captured by a CCD camera system (Fujifilm LAS 4000).

### Plant hormone quantification by HPLC-ESI-MS/MS

Methods for quantification of multiple hormones and metabolites, including ABA and its metabolites (*cis*-ABA, *trans*-ABA, ABA-GE, PA, DPA, 7’OH-ABA, and neoPA), gibberellins (GA_53_ and GA_34_), auxins/auxin conjugates (IAA, IAA-Asp, and IAA-Glu), and cytokinins (iPR, *cis*-ZR, and *cis*-ZOG) followed those previously described [53, 54].

Briefly, lyophilized seed samples were ground and a mixture of all internal standards was added to duplicate homogenized seed samples (~50 mg each), and extraction performed using acidic isopropanol. Samples were reconstituted and purified by solid phase extraction (SPE) with Sep-Pac C18 cartridges (Waters, Mississauga, ON, Canada). Subsequently, samples were injected onto an ACQUITY UPLC® HSS C18 SB column (2.1 × 100 mm, 1.8 μl) with an in-line filter and separated by a gradient elution of water containing 0.02 % formic acid against an increasing percentage of a mixture of acetonitrile and methanol (50:50, v/v). The analysis utilized the Multiple Reaction Monitoring function of the MassLynx v4.1 (Waters Inc) control software. The quality control samples and internal standard and solvent negative controls were prepared and analyzed along with samples.

## Results

### Germination profiles of white spruce seeds under different germination conditions

The mature seeds of white spruce have a relatively shallow dormancy level, and can germinate even without moist chilling. However, exposure of seeds to moist chilling led to faster and more uniform germination (Fig. 1b).

To investigate the effect of light on dormancy alleviation and germination, white spruce seeds were placed under different conditions following exposure to 21 days of moist chilling. The fastest and most homogenous germination occurred when seeds were subjected to light (an 8-h photoperiod) and a 30/20 °C temperature regime (standard germination conditions), compared to when they were kept in darkness (Fig. 1b). After the 21-day moist-chilling, subsequent seed germination under the combined conditions of 30/20 °C and an 8-h photoperiod was more successful than in constant darkness but with the same 30/20 °C temperature cycle. This was the case based on most germination parameters: dormancy index (i.e., area between germination curves of no- treatment and any treatment; 15.54 ± 2.12 vs. 7.56 ± 0.97), germination speed (i.e. the time required for 50 % germination, 8d vs. 10d), and lag time to germination (6 d vs. 7 d) (Fig. 1b). Germination capacities were similar for the two treatments at the end of the 21-day study period (96 % vs. 94 %) (Fig. 1b). Seeds subjected to a control treatment - maintaining them at 3 °C, but exposing them to an 8-h photoperiod - were unable to germinate (Fig. 1b). Regardless of the light conditions after transfer to germination temperatures (8-h photoperiod or constant darkness), germination of the population of seeds that had been subjected to moist chilling was faster and more synchronous than for the populations of seeds that had not received moist-chilling; and 21-day chilling was more beneficial than the 10-day chilling treatment (Fig. 1b). This indicates that moist-chilling has a significant effect on dormancy alleviation, and that light cues following exposure of seeds to germination temperatures facilitate germination.

### Expression of the gene *EMB32*, encoding a Late Embryogenesis Abundant (LEA) protein

Dormancy status was also investigated by monitoring the expression of the ABA-regulated gene *EMB32*, a member of the Late Embryogenesis Abundant (LEA) group. Dormancy maintenance bears some similarities to the late maturation program [55], and EMB32 and the other LEAs have a role in ensuring seed survival in the desiccated/dormant state. As such, *EMB32* can be used as a dormancy marker [56]. Indeed, during moist-chilling of white spruce seeds, the expression of this *LEA* gene was maintained at a high level, but this expression decreased very quickly when seeds were transferred to germination conditions, even as soon as six hours under standard germination conditions (Fig. 1c, 30 °C). Thus, the dormancy to germination transition began promptly when the seeds were exposed to light upon transfer to germination temperatures.

### Dynamic changes in plant hormone pathways in response to temperature cues during moist-chilling and seed germination

To investigate hormone metabolism and signalling during dormancy alleviation and germination, hormone levels and transcription of genes specifying the protein mediators of hormone metabolism and signalling were determined at the various sampling stages (Fig. 1a).

#### ABA metabolism and signalling

Biologically active *cis*-*S*(+)-ABA did not substantially change in abundance during moist-chilling itself, but decreased during subsequent germination of previously chilled white spruce seeds (Fig. 2a). The so-called “trans-ABA” is in fact a product of isomerization of natural ABA under UV light, and this did not change during the transition to germination. Generally the bioactive ABA levels were much higher than those of the ABA catabolites. From the changes in ABA metabolites it was apparent that the main ABA metabolism pathway in white spruce seeds is through 8’-hydroxylation (resulting in phaseic acid (PA), which is further reduced to dihydrophaseic acid (DPA)). Nonetheless, secondary catabolism pathways such as 7’ and 9’ hydroxylation (resulting in 7’hydroxy ABA and *neo*-PA) as well as conjugation (resulting in ABA-GE) were also represented. The various catabolites, especially PA and the 7’OH-ABA increased during moist chilling, as well as during germination of moist-chilled seeds (Fig. 2a).

Transcript abundance of *ABI3* was markedly up-regulated during the first 10 d of moist chilling, but declined to a barely detectable level at 21 d (Fig. 2c). *SnRK2.2* transcripts exhibited a similar expression pattern during the moist chilling phase (Fig. 2c). Thus, while absolute ABA levels remained constant, transcription of genes for ABA signalling components, and thereby sensitivity to ABA, started to decline during the latter part of the moist chilling phase (Fig. 2b, c). Moreover, transcripts of a putative ortholog of a negative regulator of ABI3, *CnAIP2* [31], steadily accumulated during moist-chilling and remained high during early germination (6 h). *ABI3* transcripts, were high at the mid-point during moist chilling, then declined precipitously during late moist chilling and early germination, but increased during the later stages of germination (24 and 80 h) (Fig. 2c).

Transcripts encoding *AAO3* (ABA biosynthesis enzyme) underwent few changes during moist chilling, but increased dramatically during early germination under standard conditions, followed by a decline; those for *CYP707A4* (encoding ABA 8’ hydroxylase) were not detectable (Fig. 2c). Similar to *AAO3*, transcripts encoding *CYP707A4* and *SnRK2.2* showed a significant up-regulation during the early stages when seeds were first transferred to standard germination conditions at 6 h, with transcripts declining at the later stages (Fig. 2c). An actual decline in bioactive ABA was not evident until 24 h of germination (Fig. 2a). At the seedling stage (9 d), transcripts for all of the monitored genes involved in ABA metabolism and signalling decreased to a very low level (Fig. 2c).

#### GA metabolism and signalling

**Fig. 3:**
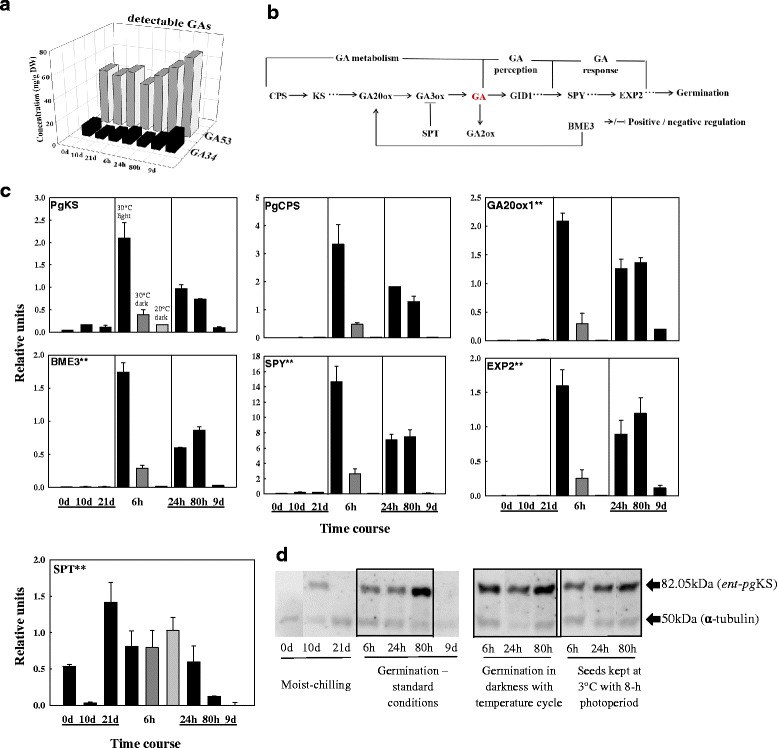
Changes in GAs and GA signalling components during the transition from dormancy to germination of white spruce seeds. **a** Profiles of the GA precursor GA_53_ and the metabolite GA_34_ as determined by UPLC/ESI-MS/MS during moist-chilling at 3 °C (0, 10, and 21 days), and during germination (6, 24 and 80 h) and seedling growth (9 d). Each data point is the average of two biological replicates. No active GAs were detected in our analysis. **b** Schematic characterization of key genes and their interplays in GA signaling cascades. Connections represent positive (arrow) and negative (block) regulation. **c** Transcript levels of GA metabolism genes and selected downstream targets during moist-chilling (0, 10, 21 d), germination (6, 24, 80 h) and seedling growth (9 d) (black bars). Also shown are previously chilled seeds placed in two control treatments - 6 h germination conditions under darkness at either 30 °C (dark grey bar) or 20 °C (light grey bar). Each data point is the average of three biological replicates. Bars indicate the SEM. Note: see Fig. 2 note for asterisks. **d** Ent-pgKS protein levels during moist-chilling, germination, and growth of white spruce seeds. Immunoblots show 30 μg of total protein extract per lane. Blots were probed with anti-KS antibody and anti-tubulin as a loading control

Of the 14 GAs that were quantified in white spruce seeds (i.e., GA_1_, _3_, _4_, _7_, _8_, _9_, _19_, _20_, _24_, _29_, _34_, _44_, _51_, and _53_), only GA_53_ and GA_34_ were present at detectable levels. GA_53_ is an early precursor in the 13-hydroxylation pathway (GA_53_ → GA_44_ → GA_19_ → GA_20_(→GA_29_) → GA_1_ → GA_8_) and leads to the formation of bioactive GA_1_ and its inactive degradation product GA_8_; GA_34_ is an inactive catabolite of biologically active GA_4_ in the non-hydroxylation biosynthetic pathway (GA_12_ → GA_15_ → GA_24_ → GA_9_(→GA_51_) → GA_4_ → GA_34_). The presence of intermediates from both biosynthesis routes suggests that both GA metabolic pathways are active in white spruce seeds during dormancy alleviation and germination. Moreover, the presence of GA_34_ suggests that GA_4_ must have been produced at earlier stages. GA_53_ of the early 13-hydroxylation pathway conducive to the formation of bioactive GA_1_ increased steadily during germination under standard conditions after seeds had received moist chilling. During moist-chilling itself, GA_53_ and GA_34_ were maintained at steady-state levels, with GA_53_ present at ~5-fold higher levels than GA_34_ (Fig. 3a). GA_34_ increased most substantially at 9 d (i.e. during seedling growth) (Fig. 3a).

Most of the GA-related genes that we monitored (those encoding mediators of GA- biosynthesis, signalling, or action; Fig. 3b) were expressed at low levels during moist-chilling (Fig. 3c). (Note that all reference genes were expressed during these times; Additional file 1: Figure S3). The expression of *SPT* (*SPATULA*), encoding a mediator of ABA- and GA- signaling cross talk, decreased to a low level at 10 d of moist chilling but exhibited a 14-fold increase at 21 d (Fig. 3c).

We also investigated transcript abundance of genes known to be positively regulated by GA as indirect indicators of the presence of active GA. The GA-regulated cell wall-modifying gene, expansin 2 (EXP2), exhibited a 15-fold up-regulation within 6 h after transfer of moist chilled seeds to standard germination conditions (Fig. 3c). The expression of other GA-related genes was also substantially increased during early germination before radicle protrusion; moderate expression occurred between 24–80 h, while at the seedling stage, the expression of all of the monitored genes was low or virtually undetectable (Fig. 3c). This is indicative of the presence of active GA during completion of germination and during very early seedling growth (seedling emergence).

Ent-*pg*KS protein levels were increased during the first 10 d of moist chilling, with a decline during the latter period of moist chilling (Fig. 3d). Upon transfer of moist-chilled seeds to germination conditions, the levels increased by 6 h (coincident with increased transcript levels; Fig. 3c). The most pronounced ent-*pg*KS protein levels were evident in seeds at 80 h under standard germination conditions; however, the control treatments indicated that either changing the light conditions or exposing seeds to germination temperatures were sufficient to trigger the increased levels of this protein (Fig. 3d).

#### Auxin metabolism and signalling

**Fig. 4:**
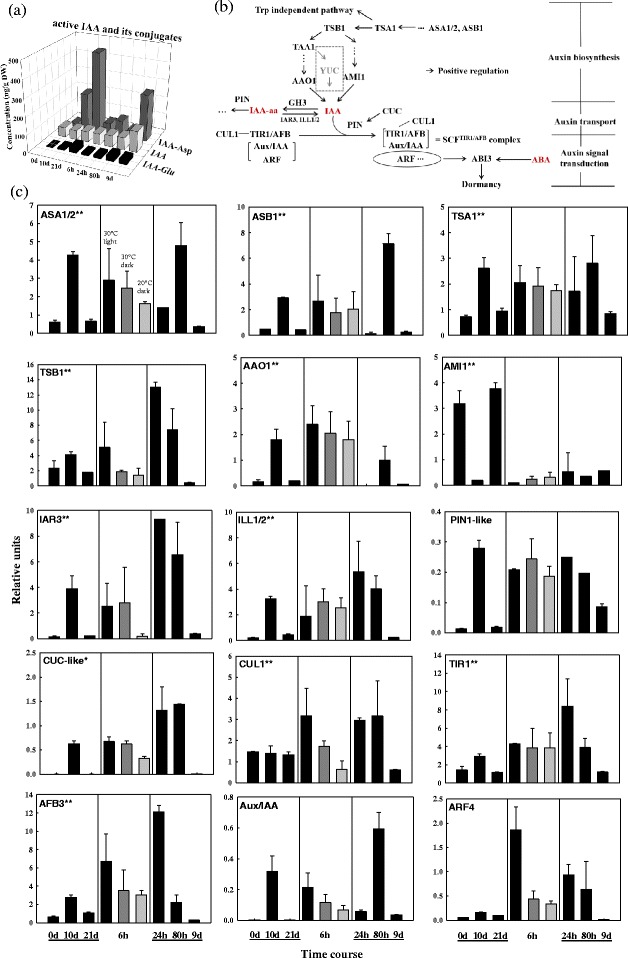
Changes in IAA, IAA conjugates, and auxin-related gene expression during the transition from dormancy to germination of white spruce seeds. **a** IAA and IAA conjugates in seeds as determined by UPLC/ESI-MS/MS during moist-chilling at 3 °C (0, 10, and 21 d) and during germination (6, 24 and 80 h) and seedling growth (9 d). Each data point is the average of two biological replicates. **b** Schematic characterization of key genes and their interplays in auxin signalling cascade. Connections represent positive (arrow) regulation. **c** Transcript levels of auxin metabolism genes, auxin signalling genes and selected downstream targets during moist-chilling (0, 10, 21 d), germination (6, 24, 80 h) and seedling growth (9 d) (black bars). Also shown are previously chilled seeds placed in two control treatments - 6 h germination conditions under darkness at either 30 °C (dark grey bar) or 20 °C (light grey bar). Each data point is the average of three biological replicates. Bars indicate the SEM. Notes: 1) see Fig. 2 note for asterisk in c; 2) no other ARF homologs (such as ARF16) and GH3 (converting active IAA to IAA-aa) homologs were found in white spruce by BLASTN

Active IAA was almost at constant levels throughout the moist chilling period, and during germination (Fig. 4a). IAA conjugates (IAA-Asp and IAA-Glu) strikingly increased over 20-fold during the 21 d of moist-chilling (Fig. 4a). These conjugated IAAs declined markedly during the first 6 h in standard germination conditions; later seedling growth was accompanied by an increase in both active and conjugated IAA (Fig. 4a).

In the auxin pathway (Fig. 4b, c), the expression of auxin biosynthesis genes (*ASA1/2*, *ASB1*, *TSA1*, *TSB1*, and *AAO1*) was highest at 10 d of moist chilling, then declined at the later stages of moist chilling (21 d). Interestingly, *AMI1*, another auxin biosynthesis gene in a parallel pathway with *AAO1*, exhibited lowest expression at 10 d of moist chilling (Fig. 4c). This suggests that auxin was actively synthesized during moist-chilling and mediators of the two pathways that synthesize auxin were separately activated at early and late moist-chilling. Likewise, *ASA1/2*, *ASB1*, *TSA1*, *TSB1*, and *AAO1* exhibited high expression levels at 6 and 80 h, while *AMI1* had a constant low expression level during germination. In Arabidopsis, there exists a third auxin biosynthesis pathway via *YUC* [57]; no homolog to the Arabidopsis *YUC* gene was found in white spruce after an extensive database search, and this auxin biosynthesis route may not exist in the seeds of this conifer species. The expression of *IAR3* and *ILL1/2*, which specify enzymes that convert conjugated IAA to active IAA, as well as expression of genes for the auxin transporters PIN1-like and CUC-like was significantly up- and then down- regulated in association with the transcript regulation of the biosynthesis genes of the *AAO1* pathway during moist chilling (Fig. 4c). In seeds placed under standard germination conditions, the genes for the auxin transporters exhibited a pattern of heightened transcript abundance during germination, and lowered expression during seedling growth (Fig. 4c).

Auxin signalling primarily depends on the TIR1/AFB auxin receptor (TAAR), Aux/IAA, and ARF4. The expression of *TIR1*, *AFB3* and *Aux*/*IAA* was significantly up and then down regulated during moist-chilling and that of *ARF4* appeared to follow the same pattern but at a lower absolute level (Fig. 4c). At 6 h in germination conditions, the expression of *TIR1*, *AFB3*, *Aux/IAA*, and *ARF4* significantly increased but only *TIR1* and *AFB3* continued to increase at 24 h. At the seedling stage, *ARF4* along with *TIR1*, *AFB3*, and *Aux*/*IAA* was expressed at a fairly low level (Fig. 4c). Likewise, transcript for CUL1, a component of SCF ubiquitin ligase complexes, was substantially produced during 80 h in germination but not during seedling growth (Fig. 4c).

### Dynamic changes of hormone signalling pathways after dormancy termination during germination and radicle protrusion

To separate the contributions of optimal germination temperature and light signalling to germination completion (i.e. radicle protrusion), two additional germination conditions in place of the standard conditions were used after seeds had received 21 days of moist chilling. As controls, seeds were not exposed to light (i.e. kept in darkness) but were exposed to either an optimal germination temperature (30/20 °C) (Fig. 1a) or a non-optimal germination temperature (constant 20 °C) (not shown in Fig. 1a). Transferring seeds to standard (i.e. optimal) germination conditions led to greater fold transcript changes than transferring seeds to the same temperature regime but keeping them in darkness. Transferring seeds to 20 °C in darkness further reduced transcript induction (Figs. 2c, 3c, and 4c). This effect was particularly obvious for the studied genes of the GA pathway.

**Fig. 5:**
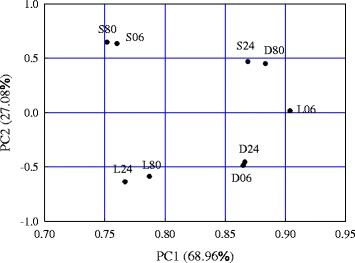
The results of principle component analysis applied to the expression of all the genes used in previous qPCR analysis in ABA and GA pathways over three different germination conditions. S06/S24/S80, D06/D24/D80, and L06/L24/L80 represent standard, darkness, and low temperature (3 °C) germination conditions corresponding to D/E/F, H/I/J, and K/L/M in Fig. 1a, respectively

PCA analysis for all studied genes in different germination conditions was conducted (Fig. 5 and Additional file 1: Figure S4). Gene expression variations (68.96 and 27.08 %) were explained by PC1 and PC2, respectively, and the PCA grouped the samples into five clusters (Fig. 5). Based on PCA analysis, we found that: 1) germination initiation (6 h) and radicle protrusion (80 h) under standard germination conditions (30/20 °C and 8-h photoperiod) were associated with similar gene expression patterns. The same was true of 6 h and 24 h in darkness with a 30/20 °C temperature cycle and of 24 and 80 h in constant low temperature (3 °C) with an 8-h photoperiod; 2) gene expression patterns at 80 h in constant darkness were similar to those at 24 h with an 8-h photoperiod; 3) six h in low temperature was associated with unique gene expression patterns. Therefore, seeds in constant darkness with temperature cycles displayed a similar expression pattern but were delayed in time, compared with those seeds placed under both optimal germination temperature and photoperiod cycles, Conversely, seeds in constant low temperature with an 8-h photoperiod exhibited different gene expression patterns at 6 h, and at 24 and 80 h, despite not completing germination (visible radicle protrusion) (Fig. 5). Taken together, temperature and light jointly promoted germination mediated by ABA, GA, and auxin pathways.

## Discussion

### Plant hormones co-ordinately respond to temperature cues

#### Moist-chilling is associated with changes in hormone flux

IAA biosynthesis was active during moist-chilling (Fig. 4c), but active IAA levels were maintained at constant levels, while conjugated IAA-Asp and IAA-Glu steadily and significantly increased (Fig. 4a). Conjugated IAAs are regarded as storage compounds, which, in seeds, are either stored to be activated by de-conjugation and serve in early seedling growth, or are used for an entry route into subsequent catabolism [58]. IAA conjugated to amino acids such as aspartate and glutamate may be largely degraded [59]. Although the function of IAA conjugates and the genes that regulate their formation is scarcely investigated, the large amount of IAA conjugates that accumulated during moist-chilling likely has biological significance. More information is required concerning the cellular distribution of the different auxin forms as well as their relative dependence on specific transport mechanisms [60].

The PIN family proteins and the recently discovered PIN-LIKES are important as IAA efflux carriers in IAA transport between the cytosol and the endoplasmic reticulum [60, 61]. We observed that, at 10 d of moist-chilling, and during subsequent germination, transcripts of *PIN1-like* and *CUC-like* were markedly up-regulated (Fig. 4c) while active IAA remained relatively constant (Fig. 4a). Auxin-induced cell expansion connected to the acidification of the cell wall, is thought to invoke an increase in the activity of the wall loosening proteins, expansins [62], which can disrupt the non-covalent bonds that form between cellulose and hemicellulose in the wall and thus promote cell expansion [63]. Despite no substantial overall increase in IAA during germination, IAA may nonetheless be redistributed within seed tissues to active areas of cell expansion due to the action of various transporters (Fig. 4c). Polar transport sets up auxin gradients in specific cell types, and such gradients can provide developmental cues during key processes including embryogenesis and root development [64, 65].

The auxin response not only depends on auxin levels and locations, but also on the specificity and strength of the TIR1-Aux/IAA and Aux/IAA-ARF interactions [45]. The decreased availability of TIR1 could lead to increased levels of free Aux/IAAs, which would combine with ARF4, thereby eventually decreasing free ARF4 levels. Hence, it is possible that prior to 10 d of moist chilling, Aux/IAA is predominantly combined with TIR1/AFB3 rather than with ARF4, and that the free ARF4 contributes to the increase of *ABI3* transcripts at 10 d (Figs. 4c and 2c), because ARFs may bind to putative auxin response elements (AuxREs) of the *ABI3* gene promoter [42]. Conversely, after 10 d, ARF4 may be more likely to interact with Aux/IAA [45], thus lowering free ARF4 and contributing to the decreasing expression of *ABI3* at 21 d (Figs. 4c and 2c). These changing interactions between components of the ABA and auxin signalling pathways may promote dormancy alleviation [42]. Our analyses were confined to monitoring transcript levels and so we can only speculate as to changes at the level of the proteins that mediate auxin and ABA action. Changes in transcript levels for the ABI3 antagonist – CnAIP2 – may also be relevant here as the *CnAIP2* promoter is exquisitely regulated by auxin.

Active ABA did not decrease during moist-chilling itself, but did decrease substantially during subsequent germination (Fig. 2a), at a time when GA_53_ of the early 13-hydroxylation pathway conducive to the formation of bioactive GA_1_, increased steadily (Fig. 3a and c). Thus an increased GA/ABA ratio, was clearly associated with germination of white spruce seeds [66]. We did not detect any bioactive cytokinin in our samples (Additional file 1: Figure S1). Cytokinin and auxin have long been known to interact antagonistically, and the past five years have seen significant advances in our understanding of the extensive crosstalk between cytokinin and various other hormones, particularly auxin (reviewed in [67]). In our study, none of bioactive free base cytokinins (zeatin, dihydrozeatin, and isopentenyladenine) was detected during moist chilling. However, the biosynthesis precursors *cis*-zeatin roboside (*cis*-ZR) and isopentenyladenine riboside (iPR) were markedly increased, while the catabolism product *cis*-zeatin-O-glucoside (*cis*-ZOG) was detected only at very low levels during moist-chilling (Additional file 1: Figure S1). This may indicate that a small amount of *cis*-zeatin was transiently produced as a result of moist chilling.

SPT (SPATULA) is thought to be involved in both ABA and GA signaling cross talk and may drive two antagonist roles in mature seeds of Arabidopsis -- ‘dormancy-promoting’ and ‘dormancy-repressing’ -- depending on the ecotype background [68, 69]. In white spruce seeds, *SPT* expression decreased to a low level at 10 d of moist chilling but exhibited a 14-fold increase at 21 d (Fig. 3c). Its role in white spruce dormancy alleviation and germination remains to be determined.

#### Germination conditions

When seeds were transferred to germination conditions, we observed remarkably strong changes in expression of our monitored genes typically by only 6 h. Transcripts encoding the GA-regulated cell wall-modifying protein expansin 2 (EXP2) were 15-fold up-regulated, indicating a strong up-regulation in GA signalling (Fig. 3c). ABA declined especially after 24 h (Fig. 2a). *ABI3* was expressed at low levels at 6 h but was up regulated at 24 h, perhaps relevant to a ‘stress sensing’ function at this critical stage (Fig. 2c). At the radicle protrusion time-point (80 h), transcripts of genes specifying auxin biosynthesis enzymes (*ASA1/2*, *ASB1*, *TSA1*, and *AAO1*), or proteins mediating conversion to active IAA (*IAR3* and *ILL1/2*), and signaling (*Aux/IAA*) were substantially produced, and these pathways may have acted in concert with those of the GA and ABA signaling pathways (Fig. 4c). Thus auxin likely also plays a pivotal role in germination of white spruce seeds.

Plants have evolved a battery of photoreceptors to sense ambient light and transduction of light signals [70]. In the control of seed dormancy and germination, phytochromes represent the most investigated photoreceptors. Phytochromes are temperature- and light-dependent in association with the GA pathway via SPT [71]. The expression of *SPT* significantly decreased at 6 h and the transcript levels were almost the same as those in the seeds exposed to light or kept in darkness. However, the seeds placed in 30 °C had a lower level of *SPT* transcripts than seeds placed in 20 °C (Fig. 3c). In white spruce, as in Arabidopsis, SPT may be a light-stable repressor of seed germination and may play a role in the germination response to temperature through temperature-sensitive changes in its transcription [68].

### Winter chilling under new climate scenarios and its effects on conifer life histories

Winter chilling is an important signal for regulating plant life histories; chilling leads to a competence for flowering through vernalization in winter annuals, and alleviates both bud and seed dormancy, allowing the onset of growth in the spring [9, 72]. It is noteworthy that climate change may ultimately result in winter shortening and an increase in the growing season length [73, 74]. In North America, the number of winter chilling days has become insufficient for bud dormancy break from 40°N southward as climate changes, leading to delayed vegetation green-up, but it has remained sufficient from 40°N northwards as earlier springs lead to an advanced green-up onset [75]. A similar geographic pattern as observed in budburst may also occur for germination in temperate regions and two possible scenarios exist depending on whether moist-chilling requirements are minimally met; namely, fast and prompt germination leading to greater recruitment (adequate chilling) or an extended germination span leading to adverse conditions during dry summers (inadequate chilling) (see Fig. 1b). Thus shorter winters may delay or advance germination [76].

On the other hand, the range of spruce trees and other conifers cover large climatic gradients while their subpopulation can be adapted to their local environments [77, 78]. These populations may draw on alternative molecular solutions to respond to local environmental conditions [79, 80]. Presumably, variations in gene expression contribute to phenotypic diversity including dormancy variation and, therefore sustain the adaptability of conifer populations [81]. As such, our results of gene expression during moist-chilling may help predict future seed recruitments in response to climate change. Finally, it is important to note that the seeds of certain other conifer species (yellow cypress, western white pine and white bark pine) exhibit much deeper dormancy at maturity than white spruce seeds. These seeds require several months of moist chilling to alleviate their dormancy, and may well be more substantially impacted by climate change, as the extended cold period is so critical for their ability to germinate.

## Conclusions

In addition to classic ABA and GA mechanisms, auxin appears to be actively involved in dormancy termination and germination of white spruce seeds. We hypothesize that auxin signalling plays a role in these processes partly by interacting with ABA signalling. This is in accordance with recent findings regarding the crosstalk of auxin and ABA in the regulation of seed dormancy in angiosperms [42]. Auxin has a dominant role in plant morphogenesis and is an inescapable player in many developmental processes and a central component of crosstalk networks. Our findings now point to auxin as a key player that likely works in conjunction with the ABA and GA signal pathways previously investigated in mechanisms underlying dormancy alleviation by chilling in conifer seeds. Our study also yields insights into the speed with which imbibed seeds can adjust their transcription to environmental conditions, as demonstrated when seeds were transferred from moist chilling to germination conditions. After only six hours in light at higher temperatures, significant changes in transcript abundance were observed.

## Additional file

Additional file 1: Table S1. Description of genes and primer pairs used for qPCR. **Figure S1.** Profiles of cytokinins and their metabolites in seeds of white spruce (as determined by UPLC/ESI-MS/MS) during moist-chilling at 3 °C (0, 10, and 21 d), and during germination (6, 24 and 80 h) and seedling growth (9 d). Each data point is the average of two biological replicates. *cis*-ZR, *cis*-Zeatin riboside; iPR, Isopentenyladenine roboside; *cis*-ZOG, *cis*-Zeatin-O-glucoside. **Figure S2.** Transcript levels of various marker genes at 6, 24, and 80 h following transfer of seeds to standard germination conditions (i.e. 8-h photoperiod and 30/20 °C) (black bars), constant darkness with a 30/20 °C cycle (light grey bars), or constant 3 °C with an 8-h photoperiod (dark grey bars). Each data point is the average of three biological replicates. Bars indicate the SEM. **Figure S3.** C(t) values for all three reference genes across studied time-points. **Figure S4.** Repeatability of hormone quantification analyses. Note: variation between two experimental replicates in four metabolites (IAA-Asp, IAA, PA, and ABA) was distinguished by colours in the panel. (DOCX 475 kb)

## Abbreviations

ABA: Abscisic acid
ABA-GE: abscisic acid glucose ester
PA: Phaseic acid
DPA: Dihydrophaseic acid
*neo*-PA: *neo*-phaseic acid
7’OH-ABA: 7’-hydroxy abscisic acid
GA: Gibberellin
GA_53/34_: Gibberellin 53 and 34
IAA: Indole-3-acetic acid
IAA-Asp: N-(Indole-3-yl-acetyl)-aspartic acid
IAA-Glu: N-(Indole-3-yl-acetyl)-glutamic acid
AAO3: Abscisic acid aldehyde oxidase 3
CYP707As: Aba 8’-hydroxylases
PYR/RCAR: Pyrbactin resistance 1-like/ regulatory component of aba receptor
PP2C: Protein phosphatase 2C
SnRK2: Sucrose nonfermenting 1 (SNF1)- Related protein kinase 2
ABI3/5: Aba insensitive 3/5
AIP2: ABI3-interacting protein 2
LEC1: Leafy cotyledon 1
ABF/AREB: Abscisic acid responsive element-binding factor
CPS: Ent-copalyl diphosphate synthase
KS: Ent- kaurene synthase
GA20ox: Gibberellin 20 oxidase
GA3ox: Gibberellin 3 oxidase
GA2ox: Gibberellin 2 Oxidase
GID1: Gibberellin insensitive dwarf 1
BME3: Blue micropylar end 3
SPY: Spindly
EXP2: Expansin A2
SPT: Spatula
ASA1/2: Anthranilate synthase component I-1/2
ASB1: Anthranilate synthase beta subunit 1
TSA1: Tryptophan synthase alpha chain 1
TSB1: Tryptophan synthase beta subunit 1
AAO1: Aldehyde oxidase 1
AMI1: Amidase 1
IAR3: IAA-alanine resistant 3 (IAA-amino acid hydrolase)
ILL1/2: IAA-leucine resistant (ILR)-like 1/2 (IAA-amino acid hydrolase)
PIN1-like: Pin formed 1-like
CUC-like: Cup-shaped cotyledon-like
CUL1: Cullin1
TIR1: Transport inhibitor response 1
AFB3: Auxin signaling F-box 3
Auxin/IAA: Auxin-induced protein 2 (AUXIN/IAA)
ARF4: Auxin responsive factor 4
