# Supplementary material for: Changes in hormone flux and signaling in white spruce (Picea glauca) seeds during the transition from dormancy to germination in response to temperature cues
Source: BMC Plant Biol. 2015 Dec 18;15:292. doi: 10.1186/s12870-015-0638-7 (PMC4683703; doi:10.1186/s12870-015-0638-7)
Supplement: Additional file 1: Table S1. — Description of genes and primer pairs used for qPCR. Figure S1. Profiles of cytokinins and their metabolites in seeds of white spruce (as determined by UPLC/ESI-MS/MS) during moist-chilling at 3 °C (0, 10, and 21 d), and during germination (6, 24 and 80 h) and seedling growth (9 d). Each data point is the average of two biological replicates. cis-ZR, cis-Zeatin riboside; iPR, Isopentenyladenine roboside; cis-ZOG, cis-Zeatin-O-glucoside. Figure S2. Transcript levels of various marker genes at 6, 24, and 80 h following transfer of seeds to standard germination conditions (i.e. 8-h photoperiod and 30/20 °C) (black bars), constant darkness with a 30/20 °C cycle (light grey bars), or constant 3 °C with an 8-h photoperiod (dark grey bars). Each data point is the average of three biological replicates. Bars indicate the SEM. Figure S3. C(t) values for all three reference genes across studied time-points. Figure S4. Repeatability of hormone quantification analyses. Note: variation between two experimental replicates in four metabolites (IAA-Asp, IAA, PA, and ABA) was distinguished by colours in the panel. (DOCX 475 kb) [file 12870_2015_638_MOESM1_ESM.docx]

**Supporting data files**

**Table S1.** Description of genes and primer pairs used for qPCR.

**Figure S1.** Profiles of cytokinins and their metabolites in seeds of white spruce (as determined by UPLC/ESI-MS/MS) during moist-chilling at 3°C (0, 10, and 21 d), and during germination (6, 24 and 80 h) and seedling growth (9 d). Each data point is the average of two biological replicates. *cis*-ZR, *cis*-Zeatin riboside; iPR, Isopentenyladenine roboside; *cis*-ZOG, *cis*-Zeatin-O-glucoside.

**Figure S2.** Transcript levels of various marker genes at 6, 24, and 80 h following transfer of seeds to standard germination conditions (i.e. 8-h photoperiod and 30/20°C) (black bars), constant darkness with a 30/20°C cycle (light grey bars), or constant 3°C with an 8-h photoperiod (dark grey bars). Each data point is the average of three biological replicates. Bars indicate the SEM.

**Figure S3.** C(t) values for all three reference genes across studied time-points.

**Figure S4.** Repeatability of hormone quantification analyses. Note: variation between two experimental replicates in four metabolites (IAA-Asp, IAA, PA, and ABA) was distinguished by colours in the panel.

**Table S1 Description of genes and primer pairs used for qPCR**

| **Gene abbreviation** | **GeneBank accession number** | **Arabidopsis homolog accession** | **Arabidopsis**  **locus description** | **BLASTN score (bits)** | **E-value** | **Identities** | **Primer pairs** | **Expected size (bp)** |
| --- | --- | --- | --- | --- | --- | --- | --- | --- |
| Peroxisomal targeting signal receptor | CO220221 |  |  | Ref. [1] | | | 5’- ATGCCTATCTGAAATGGACAC  3’- ACTGTCTATGTTTGGCAGCAC | 149 |
| Hypothetical protein | CO206996 |  |  | Ref. [1] | | | 5’- GTCGTGTGGATTGTCTCTGC  3’- ATGTATTCGAAGAGGAGGAATG | 202 |
| UBC1 | AY639585 | NM_105097 | Ubiquitin-conjugating enzyme 1 | 962 | 0.00 | 100% (477/477) | 5’- GGAACAGTGGAGTCCTGCTT  3’- CCTTGCGGTGGACTCATATT | 148 |
| EMB32 (LEA)* | DQ120067 |  | Dehydrin LEA | Ref. [2, 3] | | | 5’- GAGAACGGTGTTCTGGATGA  3’- CAGCGGTATCCCTGATGTTA | 166 |
| AAO3** | BT103270 | NM_128273 | Abscisic aldehyde oxidase 3 | 64 | 6.00E-08 | 86% (59/68) | 5’- TTCTAGCAGCATCGGTTCAC  3’- CTGCAAATAGCGCTCAACAT | 158 |
| ^†^CYP707A4^**^ | DR569557 | NM_112814 | ABA 8'-hydroxylase | 52 | 9.00E-05 | 80% (89/110) | 5’- CGAACTGGCAAAGCTACAAA  3’- ATCCGAGAGGCATGATGATT | 182 |
| SnRK2.2** | EX436780 | NM_114910 | Sucrose nonfermenting 1(Snf1)-related protein kinase 2.2 | 202 | 3.00E-50 | 80% (419/522) | 5’- CGTGACTTGAAGCTGGAAAA  3’- CCACAGGACCATACATCTGC | 197 |
| ^†††^ABI3* | BT102260 | AJ131113 | ABA insensitive 3 | 321 | 1.00E-85 | 87% (288/330) | 5’- ACGTTGGCAATCTAGGAAGG  3’- CGCCAGTATTTTCAAGCAGA | 186 |
| ^††††^AIP2* | BT118051 | AY268951 | ABI3 interacting protein 2 | 339 | 3.00E-91 | 80% (590/732) | 5’- ATGTGAAGCCCCTTTCATTC  3’- AGAAGCGCCGATAAACTTTG | 172 |
| *Pg*KS | GU144565 |  | *Ent*-kaurene synthase | Ref. [4] | | | 5’- ACATGGAAAATGCAGAACCA  3’- CTCTCTTGCAGCCTTGAATG | 174 |
| *Pg*CPS | ES262766 |  | *Ent*-copalyl diphosphate synthase | Ref. [4] | | | 5’- CTTGGTATCGCCCGATATTT  3’- GTGCGAACGAAGAAGTCTGA | 153 |
| GA20ox1** | DR575289 | NM_118674 | Gibberellin 20 oxidase 1 | 54 | 2.00E-05 | 89% (42/47) | 5’- GAGAAATAACGCCCACGAAT  3’- GGCCATAGATTTGCCCTAAA | 196 |
| BME3** | DR570635 | NM_115338 | Blue micropylar end 3 | 52 | 9.00E-05 | 83% (65/78) | 5’- GTATTCGGGCAGAAGCCTAC  3’- GGAATCTGAACCCCTGAAGA | 199 |
| SPY** | EX309351 | NM_111987 | Spindly | 105 | 2.00E-20 | 78% (396/377) | 5’- AGACTCGTTGGCAGATCCTT  3’- TGAAGCTTCCAAAGGTGATG | 158 |
| EXP2** | BT104733 | NM_120611 | Expansin A2 | 60 | 2.00E-07 | 91% (42/46) | 5’- GGCAAAGCAACTCCTACCTC  3’- CATCTGCATTCGAGCTGTCT | 170 |
| SPT** | EX439688 | NM_119857 | SPATULA | 68 | 1.00E-09 | 80% (118/146) | 5’- CGCAAGAAGATTCTGGTGAA  3’- GGTACTCGATTGCTTCGTCA | 204 |

(CONTINUED)

| **Gene abbreviation** | **GeneBank accession number** | **Arabidopsis homolog accession** | **Arabidopsis**  **locus description** | **BLASTN score (bits)** | **E-value** | **Identities** | **Primer pairs** | **Expected size (bp)** |
| --- | --- | --- | --- | --- | --- | --- | --- | --- |
| ASA1/2** | BT105301 | NM_001203302 | Anthranilate synthase component I-1/2 | 150 | 4E-31 | 71% (198/280) | 5’- cctatgttcctgggatgctt  3’- cgaaaagactgtcggattca | 153 |
| ASB1** | BT110778 | AY099834 | Anthranilate synthase beta subunit 1 | 404 | 2.00E-100 | 74% (429/576) | 5’- aaattcagccattcccaaag  3’- gctcgggtttaccatgttct | 163 |
| TSA1** | BT112370 | NM_115321 | Tryptophan synthase alpha chain 1 | 200 | 7.00E-45 | 69% (329/478) | 5’- cctatgttcctgggatgctt  3’- cgaaaagactgtcggattca | 153 |
| TSB1** | BT109977 | NM_124862 | Tryptophan synthase beta subunit 1 | 802 | 0.00E+00 | 77% (721/934) | 5’- gaggtggttcaaatgcaatg  3’- tgcccgtcttcatcttgtag | 184 |
| AAO1** | BT103270 | NM_180718 | Aldehyde oxidase 1 | 222 | 1.00E-50 | 72% (282/393) | 5’- ttctagcagcatcggttcac  3’- ctgcaaatagcgctcaacat | 158 |
| AMI1** | BT113550 | NM_100769 | Amidase 1 | 206 | 3.00E-46 | 70% (305/435) | 5’- cagtggcaaaaggctatcaa  3’- cagtcccccttctcaaatgt | 187 |
| IAR3** | BT106976 | NM_104055 | IAA-alanine resistant 3 (IAA-amino acid hydrolase) | 332 | 1.00E-80 | 68% (598/873) | 5’- gagggagcattggaaaatgt  3’- ttgaatgttgtgggattgct | 166 |
| ILL1/2** | BT106591 | NM_125049(8) | IAA-leucine resistant (ILR)-like 1/2 (IAA-amino acid hydrolase) | 338 | 3.00E-82 | 70% (504/723) | 5’- attgattgccttccaacaca  3’- tctcccagacgagtgtcaag | 154 |
| PIN1-like | DR565243.1 | FJ031883 | Pin formed 1-like | Ref. [5] | | | 5’- tctggcattcgctttaactc  3’- atacacccacccgaaaatct | 179 |
| CUC-like | BT102493.1 | HM638414 | Cup-shaped cotyledon-like | Ref. [6] | | | 5’- accatgtccagcaacctcct  3’- tatggagctgggcctgattt | 108 |
| CUL1** | BT115619 | NM_001203732 | cullin 1, a component of SCF ubiquitin ligase complexes | 846 | 0.00E+00 | 76% (800/1051) | 5’- cccttgcatgtgcaaaatac  3’- ccttgtccacatcctcaatg | 171 |
| TIR1** | BT107385 | NM_116163 | Transport inhibitor response1 | 276 | 3.00E-65 | 68% (554/819) | 5’- aaatgcagcaaatgaacagc  3’- ttgcagagaatgttgccttc | 187 |
| AFB3** | BT110362 | NM_101152 | auxin signalling F-box 3 | 130 | 1.00E-25 | 66% (328/500) | 5’- tgattggttgagctgctttc  3’- ttgtggggctctcaacataa | 190 |
| Auxin/IAA* | EX428820 | AY289601 | auxin-induced protein 2 (auxin/IAA2) | Ref. [7] | | | 5’- gaagtcatggactccaccag  3’- ctgactaggagatgccgaaa | 156 |
| ARF4 | CO256727 |  | Auxin responsive factor 4 | Ref. [1] | | | 5’- ATTGCCCCGTTAAGTCTAATG  3’- CCTTTTCCCCTGATTGTTGAG | 171 |

* the gene has been annotated in other conifer species and the homolog accession number is from conifers instead of from Arabidopsis;

** the gene has only been annotated in Arabidopsis and related references have been displayed in the space of five to seven columns;

^†^ with reference to [8, 9];

^††^ with reference to [10];

^†††^ with a reference to [11];

^††††^ with a reference to [12, 13].

**Figure S1**

**Germination**

**Moist-chilling**

**Figure S2**

6h 24h 80h

**Relative units**

**Time course**

6h 24h 80h

6h 24h 80h

**Figure S3**

**Time course**

30°C 30°C 20°C

light dark dark

light

**0d 10d 21d 6h 24h 80h 9d**

**C (t) value**

**Figure S4**

**Time course**

**Concentration (ng/g DW)**

**References**

1. Friedmann M, Ralph SG, Aeschliman D, Zhuang J, Ritland K, Ellis BE, Bohlmann J, Douglas CJ: **Microarray gene expression profiling of developmental transitions in Sitka spruce (*Picea sitchensis*) apical shoots**. *J Exp Bot* 2007, **58**(3):593-614.

2. Dong JZ, Dunstan DI: **Expression of abundant mRNAs during somatic embryogenesis of white spruce [*Picea glauca* (Moench) Voss]**. *Planta* 1996, **199**(3):459-466.

3. Xia JH, Kermode AR: **Analyses to determine the role of embryo immaturity in dormancy maintenance of yellow cedar (*Chamaecyparis nootkatensis*) seeds: synthesis and accumulation of storage proteins and proteins implicated in desiccation tolerance**. *J Exp Bot* 1999, **50**(330):107-118.

4. Keeling CI, Dullat HK, Yuen M, Ralph SG, Jancsik S, Bohlmann J: **Identification and functional characterization of monofunctional *ent*-copalyl diphosphate and *ent*-kaurene synthases in white spruce reveal different patterns for diterpene synthase evolution for primary and secondary metabolism in gymnosperms**. *Plant Physiol* 2010, **152**(3):1197-1208.

5. Hakman I, Hallberg H, Palovaara J: **The polar auxin transport inhibitor NPA impairs embryo morphology and increases the expression of an auxin efflux facilitator protein PIN during *Picea abies* somatic embryo development**. *Tree Physiol* 2009, **29**(4):483-496.

6. Larsson E, Sundstrom JF, Sitbon F, von Arnold S: **Expression of *PaNAC01*, a *Picea abies CUP-SHAPED COTYLEDON* orthologue, is regulated by polar auxin transport and associated with differentiation of the shoot apical meristem and formation of separated cotyledons**. *Ann Bot* 2012, **110**(4):923-934.

7. Goldfarb B, Lanz-Garcia C, Lian ZG, Whetten R: **Aux/IAA gene family is conserved in the gymnosperm, loblolly pine (*Pinus taeda*)**. *Tree Physiol* 2003, **23**(17):1181-1192.

8. Kushiro T, Okamoto M, Nakabayashi K, Yamagishi K, Kitamura S, Asami T, Hirai N, Koshiba T, Kamiya Y, Nambara E: **The Arabidopsis cytochrome P450 CYP707A encodes ABA 8′-hydroxylases: key enzymes in ABA catabolism**. *EMBO J* 2004, **23**(7):1647-1656.

9. Saito S, Hirai N, Matsumoto C, Ohigashi H, Ohta D, Sakata K, Mizutani M: **Arabidopsis CYP707As encode (+)-abscisic acid 8'-hydroxylase, a key enzyme in the oxidative catabolism of abscisic acid**. *Plant Physiol* 2004, **134**(4):1439-1449.

10. Klimaszewska K, Pelletier G, Overton C, Stewart D, Rutledge RG: **Hormonally regulated overexpression of *Arabidopsis* WUS and conifer LEC1 (*CHAP3A*) in transgenic white spruce: implications for somatic embryo development and somatic seedling growth**. *Plant Cell Rep* 2010, **29**(7):723-734.

11. Zeng Y, Raimondi N, Kermode AR: **Role of an ABI3 homologue in dormancy maintenance of yellow cedar seeds and in the activation of storage protein and *Em* gene promoters**. *Plant Mol Biol* 2003, **51**(1):39-49.

12. Zhang XR, Garreton V, Chua NH: **The AIP2 E3 ligase acts as a novel negative regulator of ABA signaling by promoting ABI3 degradation**. *Gene Dev* 2005, **19**(13):1532-1543.

13. Zeng Y, Zhao T, Kermode AR: **A conifer ABI3-interacting protein plays important roles during key transitions of the plant life cycle**. *Plant Physiol* 2013, **161**(1):179-195.
